# Anti-senescence Effects of Nanovesicles Derived from Cluster of Differentiation-146-Positive Tonsil Mesenchymal Stem Cells via Modulation of the Tumor Protein 53 Pathway

**DOI:** 10.34133/bmr.0371

**Published:** 2026-05-21

**Authors:** Dohyun Kim, Hong Bae Jeon, Kyu-Sik Shim, Jeong Su Park, Jeong-Kee Yoon, Jun Hee Lee, Jeong-Hui Park, Jang-Hyun Park, Jieun Kim, Jinseo Yoon, Jung Bok Lee, Wooyeol Baek

**Affiliations:** ^1^ PLCOskin Co., Ltd, Seoul 03721, Republic of Korea.; ^2^ Department of Medicine, Yonsei University Graduate School, Seoul 07322, Republic of Korea.; ^3^ Department of Plastic and Reconstructive Surgery, Dankook University Hospital, Dankook University College of Medicine, Cheonan, Chungnam 31116, Republic of Korea.; ^4^ Department of Systems Biotechnology, Chung-Ang University, Anseong, Gyeonggi 17546, Republic of Korea.; ^5^ Institute of Tissue Regeneration Engineering (ITREN), Dankook University, Cheonan, Chungnam 31116, Republic of Korea.; ^6^ Department of Biomaterials Science, College of Dentistry, Dankook University, Cheonan, Chungnam 31116, Republic of Korea.; ^7^ Department of Biological Science, Research Institute of Women’s Health, Brain Korea 21 Project, Sookmyung Women’s University, Seoul 04310, Republic of Korea.; ^8^ Department of Biological Science, Sookmyung Women’s University, Seoul 04310, Republic of Korea.; ^9^ Department of Plastic and Reconstructive Surgery, Institute for Human Tissue Restoration, Severance Hospital, Yonsei University College of Medicine, Seoul 07322, Republic of Korea.

## Abstract

Aging greatly contributes to the progressive dysfunction of tissues and organs that accompany the aging process. Recently, nanovesicles derived from tonsil mesenchymal stem cells (TMSC-NVs) were investigated for their potential to reverse cellular senescence. However, the molecular mechanisms through which TMSC-NVs prevent senescence remain unclear. In the present study, we focused on Cluster of Differentiation 146 (CD146), a surface marker used to identify specific MSC subgroups and examined the anti-senescence effects of CD146-positive TMSC-NVs (CD146^+^ TMSC-NVs). To evaluate the antisenescence effects of the CD146^+^ TMSC-NVs, passage-associated cellular senescence in vitro human dermal fibroblast model, ultraviolet-B-induced photoaging ex vivo model, and intrinsic aging in vivo mice model were assessed. Microarray analysis, immunofluorescence staining, and miRNA profiling demonstrated the anti-senescence mechanism of CD146^+^ TMSC-NVs through modulation of the p53 pathway, thereby effectively suppressing cellular senescence and matrix metalloproteinase activity and improving extracellular matrix remodeling. These results indicate the anti-senescence effects of CD146^+^ TMSC-NVs for therapeutic approach.

## Introduction

The global aging population is expected to increase to unprecedented levels due to decreasing fertility rates and increasing life expectancy. This demographic change is related to an increased prevalence of age-related diseases, imposing a substantial burden on society, the healthcare system, and the economy. Half of the global population growth is expected to stem from an increase in the number of individuals aged ≥60 years from 2010 to 2050. Furthermore, the population of those ≥60 years of age is expected to double, whereas the number of individuals <60 years of age is likely to decrease [1,2]. Aging is predominantly characterized by cognitive decline, cardiovascular impairment, tissue degeneration, and metabolic changes that influence individuals at varying rates. Additionally, the senescence of the immune system substantially contributes to age-related diseases, which are the leading causes of death after 65 years of age. These diseases include arthritis, cardiovascular disease, cancer, dementia, osteoporosis, diabetes, hypertension, neuropathy, stroke, obesity, and depression [3,4].

Aging is characterized by 9 key hallmarks: cellular senescence, mitochondrial dysfunction, epigenetic changes, dysregulated nutrient sensing, genomic instability, altered intercellular communication, telomere shortening, and stem-cell exhaustion [5–10]. Stem cells play a pivotal role in the regulation and progression of aging. Maintaining a delicate and dynamic balance within the stem-cell niche is important for supporting stem-cell differentiation into diverse progenitor cells or lineages, which could potentially slow the aging process [11].

Studies have increasingly focused on the regenerative potential of exosomes, which are nanosized biomimetic vesicles, considering that the limitations in the clinical application of stem cells have become more apparent. Stem-cell-derived exosomes are popular because of their capacity to modulate the immune system, stimulate cell proliferation, increase angiogenesis, inhibit apoptosis, decrease oxidative stress, and prevent functional decline [3,12]. Exosomes have been extensively investigated for their potential use in anti-senescence therapies. Previous studies have revealed that exosomes derived from umbilical cord mesenchymal stem cells (MSCs) can be absorbed by human skin, thereby improving the synthesis of type I collagen (COL1) and elastin, which are 2 key components of skin rejuvenation. These findings indicate promising cosmetic and therapeutic applications of exosome-based treatments [13].

Exosomes have considerable therapeutic potential; however, they present several limitations, including low efficiency, prolonged procedures, and the need for specialized technical expertise. Additionally, ensuring size uniformity, content consistency, and high yield during labor-intensive and time-consuming production processes remain significant challenges [14]. To overcome these hurdles, exosome-mimetic nanovesicles (NVs), which demonstrate characteristics similar to those of exosomes, have been used in skin tissue regeneration, wound healing, and anti-senescence therapies. Kim et al. [15] revealed that NVs derived from tonsil mesenchymal stem cells (TMSC-NVs) mitigate cellular senescence caused by oxidative stress. However, the molecular mechanisms through which TMSC-NVs control cellular senescence have been unclear.

Cluster of Differentiation 146 (CD146), a cell adhesion molecule, is expressed on various epithelial cells, activated T cells, and dendritic cells [16]. It appears to be a valuable surface marker for determining specific MSC subpopulations. The CD146^+^ MSC subgroup has been found to exhibit improved biological activity and therapeutic potential in regenerative medicine [17]. Previous studies have revealed that CD146^+^ MSCs have strong immunomodulatory properties [18]. Li et al. [19] demonstrated that CD146^+^ umbilical cord MSCs possess superior regenerative capabilities compared to CD146^−^ MSCs in the context of premature ovarian failure. However, the therapeutic potential of NVs derived from CD146^+^ tonsil-derived MSCs (CD146^+^ TMSC-NVs) remains unclear.

In this study, we investigated the therapeutic roles of CD146^+^ TMSC-NVs in cell proliferation and cellular senescence using in vitro analysis of a passage-associated cellular senescence model. Additionally, an ultraviolet B (UVB)-induced photoaging model was utilized to assess the therapeutic potential of CD146^+^ TMSC-NVs. Histological analysis of the skin, brain, and liver were conducted following the intraperitoneal administration of CD146^+^ TMSC-NVs in an intrinsic aging mouse model. Microarray analysis and miRNA profiling were conducted to investigate the mechanisms underlying these anti-senescence effects.

## Materials and Methods

### Isolation of TMSCs

TMSCs were isolated from human tonsil tissue after tonsillectomy as previously described [15]. The tonsil tissues were rinsed with phosphate-buffered saline (PBS; Welgene, Seoul, Republic of Korea) supplemented with 2% antibiotics–antimycotics (Thermo Fisher Scientific, MA, USA). The tissues were gently minced and digested using 210 U/ml of collagenase I (Gibco, New York, NY, USA) and 4 kU/ml of DNase I (Sigma-Aldrich, St. Louis, MO, USA) in low-glucose Dulbecco’s modified Eagle’s medium (DMEM; Gibco, New York, NY, USA) at 37 °C for 90 min. The digested mixture was filtered through a 40-μm strainer and centrifuged at 1,300 rpm for 3 min. The cell pellet was washed twice with DMEM and cultured in DMEM containing 10% fetal bovine serum (Gibco) and 1% antibiotics–antimycotics at 37 °C and under 5% CO_2_. The medium was replaced every 2 d.

### Separation of CD146^+^ TMSCs via magnetic-activated cell sorting

TMSCs were separated into CD146^+^ TMSCs and CD146^−^ TMSCs at passages 2 to 4 using magnetic-activated cell sorting (Miltenyi Biotec, Bergisch Gladbach, Germany). The TMSCs were washed with PBS, trypsinized, and incubated with FcR Blocking Reagent and CD146 MicroBeads (Miltenyi Biotec) at 4 °C for 20 min. CD146^+^ TMSCs and CD146^−^ TMSCs were then separated using large-scale cell separation columns (LS Columns; Miltenyi Biotec). The separated cells were immediately used to fabricate NVs.

### Flow cytometry

To evaluate the population of CD146^+^ TMSCs, flow cytometry was assessed. Cells were washed with PBS and trypsinized for 2 min at 37 °C. Cells were incubated with phycoerythrin-conjugated anti-CD146 antibody (BD Biosciences, CA, USA) for 2 h at 4 °C. Soluble antibody was discarded with centrifugation at 1,000 rpm for 3 min. The population of CD146^+^ TMSCs was measured by flow cytometry (BD FACS Verse I; Becton Dickinson, NJ, USA). The area of fluorescence-unstained TMSCs was first evaluated and then the population of fluorescence-labeled CD146^+^ TMSCs was measured.

### Fabrication of NVs from CD146^+^ TMSCs

NVs from CD146^+^ TMSCs (CD146^+^ TMSC-NVs) were manufactured via the serial extrusion of cells through 10-, 5-, and 0.4-μm filters using a mini extruder (Avanti Polar Lipids, Alabaster, AL, USA). The characteristics of each NV, such as the expression of exosome-like surface markers (CD81/CD63/CD9) and size distribution, were verified using the Exoview R100 platform (Boston, MA, USA). Morphology was assessed using transmission electron microscopy (JEM-1011; JEOL, Tokyo, Japan). The concentrations of the NV proteins were quantified using the Micro BCA Protein Assay Kit (Thermo Fisher Scientific). The surface charge (zeta potential) of CD146^+^ TMSC-NVs was evaluated using a particle-size and zeta-potential analyzer (ELS-Z1000; Otsuka Electronics, Osaka, Japan).

### Cell culture

Human dermal fibroblasts (HDFs) were obtained from the American Type Culture Collection (ATCC, Manassas, VA, USA). The cells were maintained in DMEM supplemented with 10% fetal bovine serum and 1% antibiotics–antimycotics at 37 °C in a 5% CO_2_ environment. The medium was replaced every 2 d. Cellular senescence was induced through repeated cell passaging, with passage 3 cells classified as “young HDFs” and passage 15 cells as “old HDFs”. The cell population was assessed using the Cell Counting Kit-8 assay, following the manufacturer’s instructions (Dojindo, Kumamoto, Japan). Optical density was measured at 450 nm.

### Senescence-associated β-galactosidase assay

The senescence-associated β-galactosidase (SA-β-gal) assay was conducted using a cellular senescence staining kit (Cell Biolabs, San Diego, CA, USA). The cells were fixed with 0.25% glutaraldehyde solution at room temperature for 5 min. The supernatant was discarded after fixation, and each well was gently washed 3 times with fresh PBS. X-gal staining working solution was then added, and the cells were incubated overnight at 37 °C. The supernatant was discarded, and the cells were gently washed twice with PBS. Quantitative data were obtained by documenting the colorization ratio of the senescent cells.

### Quantitative real-time polymerase chain reaction

Total RNA was extracted using TRIzol Reagent (Ambion, Waltham, MA, USA) to confirm RNA expression in the cells. The cells were treated with 500 μl of TRIzol Reagent 6 d after treatment with 50 μg/ml of each NV. The cells were further treated with 100 μl of chloroform (Sigma-Aldrich) and incubated on ice for 15 min. The samples were centrifuged at 13,000 rpm for 15 min, and a transparent supernatant was obtained. The supernatant was treated with the same volume of isopropanol (Sigma-Aldrich), incubated at room temperature for 15 min, and centrifuged at 1,300 rpm for 15 min for RNA precipitation. The total RNA concentration was measured using a NanoDrop 2000 (Thermo Fisher Scientific), and 1 μg of total RNA was used for complementary DNA (cDNA) synthesis using the PrimeScript RT Reagent (RR037A; Takara Bio Inc., Shiga, Japan) following the manufacturer’s recommendations. Power SYBR Green PCR Master Mix (Applied Biosystems, San Francisco, CA, USA) was used for quantitative real-time polymerase chain reaction (qPCR), and the reaction was measured using the StepOnePlus real-time PCR system (Applied Biosystems). The sequences of the primer pairs are shown in Table S1. The expression of glyceraldehyde 3-phosphate dehydrogenase (GAPDH) was used to normalize amplification.

### Ex vivo analysis of a UVB-induced photoaging model of human skin

The ex vivo analysis was approved by the Institutional Review Board of Yonsei University Health System, Severance Hospital (4-2023-0160). Human skin was obtained from surgical procedures, gently washed in PBS containing 2% antibiotics–antimycotics, and trimmed to remove the hypodermis. Semisolid media were prepared by mixing 2% agarose and culture media at a 1:5 ratio and dispensed into each well of a 6-well plate. Human skin sections measuring 10 × 10 mm were placed in the semisolid media, followed by 300 mJ/cm^2^ of UVB irradiation daily for 3 d in the presence of 50 or 100 μg/ml of CD146^+^ TMSC-NVs. After 6 d, the tissues were fixed with 4% paraformaldehyde, embedded in paraffin, and processed for histological analysis.

### Cellular internalization of NVs

To visualize the cellular internalization of NVs, purified CD146^+^ TMSC-NVs were labeled using a PKH26 Red Fluorescent Cell Linker Kit (Sigma-Aldrich) according to the manufacturer’s instructions. Briefly, CD146^+^ TMSC-NVs and vehicle were diluted in Diluent C and incubated with PKH26 dye for 5 min at room temperature. The reaction was quenched by adding an equal volume of 1% bovine serum albumin in PBS. To remove unbound excess dye, the mixture was transferred to an Amicon Ultra-15 Centrifugal Filter Unit (100 kDa molecular weight cutoff; Millipore, Billerica, MA, USA). The labeled NVs were washed 3 times by adding PBS and centrifuging at 5,000 *g* for 15 min at 4 °C until the excess dye was completely removed. The final labeled NVs were recovered from the filter sleeve and resuspended in PBS for further use. Old HDFs were treated with 50 and 100 μg/ml of PKH26-labeled CD146^+^ TMSC-NVs for 24 h. Following incubation, the cells were washed 3 times with PBS to remove non-internalized NVs and fixed with 4% paraformaldehyde. For visualization, the actin cytoskeleton was stained with Alexa Fluor 488-conjugated Phalloidin (Abcam, Cambridge, UK) and nuclei were counterstained with Hoechst 33342 (Thermo Fisher Scientific). Fluorescence images were acquired using a confocal microscope. The efficiency of NV uptake was quantified by measuring the mean fluorescence intensity (MFI) of the PKH26 signal using ImageJ software (NIH, USA).

### Preparation of intrinsic aging mice and the intraperitoneal administration of CD146^+^ TMSC-NVs

All the experimental animals in the present study were purchased from Central Lab. Animal Inc. (Seoul, Republic of Korea). The Institutional Animal Care and Use Committee of Yonsei University approved the in vivo analysis (IACUC no. 2022-0096). Nine female C57BL/6 mice, two 2-month-old mice, and seven 26-month-old mice were used to assess the effects of the NVs. All the mice were pathogen free and maintained under the same environmental conditions without any differences in food intake. The mice were housed in an animal facility and treated according to the guidelines of the Yonsei Laboratory Animal Research Center. TMSC-NVs were intraperitoneally administered upon the completion of the NV preparation. Old mice were randomly categorized into 4 groups following the injections: control group (*n* = 2), TMSC-NVs (*n* = 2), CD146^+^ TMSC-NVs (*n* = 2), and CD146^−^ TMSC-NVs (*n* = 1). Then, 150 μg/300 μl of each NV were intraperitoneally administered twice, with a 7-d interval between injections. The old mice in the control group were administered 300 μl of PBS only. Young mice were included for integrated comparisons with the other groups. All the mice were euthanized 2 weeks after terminal administration, and their skin, brains, and livers were obtained for subsequent experimental analysis. These tissues were fixed in 4% formaldehyde for more than 48 h at 4 °C. They were then cut into 4-μm slices following the paraffin embedding and stained with hematoxylin and eosin and Masson’s trichrome for histological evaluations using standard procedures. Images were observed using a light microscope (Olympus, Tokyo, Japan).

### Immunohistochemistry staining

The samples were fixed in 4% paraformaldehyde at 4 °C before paraffin embedding. The paraffin-embedded tissues were sectioned into 4-μm-thick sections using a microtome. After deparaffinization with xylene and hydration with graded ethanol, heat-induced antigen retrieval was performed using 10 mM sodium citrate buffer (Sigma-Aldrich) supplemented with 0.05% Tween 20 (Junsei, Tokyo, Japan). Peroxidase was blocked with BLOXALL Endogenous Blocking Solution (Vector Laboratories, Inc., CA, USA) at room temperature for 10 min after antigen retrieval. To prevent the nonspecific binding of antibodies, 1.5% normal horse serum (Vector Laboratories) was added for 30 min at room temperature. Primary anti-COL1, anti-type III collagen (COL3), anti-matrix metalloproteinase 13 (MMP13), anti-involucrin, anti-fillagrin, anti-loricrin, anti-elastin, anti-phospho-Tau (p-Tau), anti-amyloid beta (Aβ), anti-alpha-smooth muscle actin (α-SMA), and anti-perilipin-1 antibodies were incubated with the respective samples overnight at 4 °C. All antibodies were used at a 1:200 dilution and were purchased from Abcam (Cambridge, UK). Biotinylated Goat anti-Rabbit IgG Antibody (H+L) (Vector Laboratories) and VECTASTAIN Elite ABC-HRP Reagent (Vector Laboratories) were used for 30 min each after washing the samples with 0.05% Tween 20 in PBS. 3,3′-Diaminobenzidine (DAB) staining was performed using a DAB Substrate Kit (Vector Laboratories) to detect the proteins of interest. Mayer’s hematoxylin solution (Abcam) was used for nuclear counterstaining. Images were captured using an optical microscope (Olympus) with at least 3 independent replicates per group. The slides were analyzed in full or sampled using small boxes randomly scattered across the tissue area. The color deconvolution method was adopted using Fiji ImageJ open software to quantify the staining [20].

### Western blotting

The samples were homogenized in a lysis buffer containing protease inhibitor and phosphatase inhibitors (Thermo Fisher Scientific). The homogenates were incubated at 90 °C for 2 h, followed by centrifugation at 12,000 *g* for 20 min at 4 °C. The protein concentration was determined using a Pierce BCA Assay Kit (Thermo Fisher Scientific). Next, 40 μg of protein was separated via sodium dodecyl sulfate–polyacrylamide gel electrophoresis for each sample. The proteins were transferred onto nitrocellulose membranes (Bio-Rad, Hercules, CA, USA), which were then incubated overnight at 4 °C with primary antibodies p-Tau (1:2,000; Thermo Fisher Scientific), Aβ (1:2,000, Thermo Fisher Scientific), p53 (1:2,000; CST, Danvers, MA, USA), caspase-3 (1:2,000; Novus Biologicals, Centennial, CO, USA), β-actin (1:5,000; Abcam), and GAPDH (1:5,000; Santa Cruz Biotechnology, Dallas, TX, USA). The membranes were further incubated with horseradish peroxidase-conjugated secondary antibodies (1:5,000; CST) at room temperature for 2 h. Protein bands were visualized using the SuperSignal West Pico Plus Chemiluminescent Substrate (Thermo Fisher Scientific), and images were captured using an iBright imaging system (Thermo Fisher Scientific). The level of each protein was quantified by normalization using β-actin or GAPDH.

### Microarray analysis

The Affymetrix Whole Transcript Expression Array was conducted by Macrogen, Inc. (Seoul, Republic of Korea) following the manufacturer’s protocol for the GeneChip Whole Transcript PLUS Reagent Kit. All the NVs were treated for 12 h to demonstrate the gene modulation and anti-senescence effects of the CD146^+^ TMSC-NVs. All samples were prepared and isolated in triplicates. RNA purity and integrity were evaluated using an ND-2000 Spectrophotometer (NanoDrop, Wilmington, DE, USA) and an Agilent 2100 Bioanalyzer (Agilent Technologies, Palo Alto, USA). The GeneChip Whole Transcript Amplification Kit was used to synthesize cDNA for microarray analysis. The sense cDNA was fragmented and biotin labeled with terminal deoxynucleotidyl transferase using a GeneChip WT Terminal labeling kit. Next, 5.5 μg of labeled target cDNA was hybridized to the Affymetrix GeneChip Mice Clariom S Array at 45 °C for 16 h. The hybridized arrays were washed, stained on a GeneChip Fluidics Station 450, and scanned using a GCS3000 Scanner (Affymetrix, CA, USA). The data were processed with the Affymetrix GeneChip Command Console Software (AGCC; Thermo Fisher Scientific). Gene Ontology and Kyoto Encyclopedia of Genes and Genomes (KEGG) pathway enrichment analysis were conducted to determine differentially expressed genes (DEGs) at the biological functional level [21]. DEGs associated with aging were selected from the Database for Annotation, Visualization, and Integrated Discovery [22]. A *P* value of <0.05 was considered to indicate a statistically significant difference [23]. The expression of the selected genes was subjected to hierarchical clustering and heatmap analysis.

### miRNA profiling

To demonstrate the components within NVs, miRNA profiling was conducted by Macrogen, Inc. following the manufacturer’s protocol for the TruSeq Stranded mRNA Library Prep Kit. All samples were prepared and isolated in triplicates. RNA purity and integrity were evaluated using an ND-2000 Spectrophotometer (NanoDrop). miRNA was selected based on differential expression and biological relevance to aging-related processes including extracellular matrix (ECM) remodeling, oxidative stress, and cellular senescence and unique molecular signature. The expression of miRNA was normalized as transcripts per million (TPM), followed by log_2_-transformation after adding a pseudocount of 1, denoted as “log_2_(TPM + 1)” for heatmap visualization as described by Yang and Gorski [24].

### Immunofluorescence staining

Cells were seeded on 12-mm glass coverslips and, following treatment with vehicle or various NVs, were washed with PBS and fixed with 4% paraformaldehyde for 15 min at room temperature. The cells were permeabilized with 0.1% Triton X-100 in PBS (PBS-T) for 15 min. After washing, nonspecific binding was blocked by incubating the cells with 5% bovine serum albumin in PBS-T for 30 min. The cells were then incubated with primary antibodies against phosphorylated tumor protein 53 (p-p53, Ser15) (1:50; Cell Signaling Technology, Danvers, MA, USA) and p21 (1:100; Santa Cruz Biotechnology) at 4 °C overnight. Following 3 washes with PBS-T, the cells were incubated with fluorescence-conjugated secondary antibodies for 1 h at room temperature. Nuclei were counterstained with Hoechst 33342 (Thermo Fisher Scientific) for 20 min. After a final wash and mounting, fluorescence signals were visualized using a confocal laser scanning microscope (ZEISS, LSM 900). The percentage of positive cells was quantified using ImageJ software.

### Statistical analysis

All experiments were performed in triplicates. All graphs were drawn using GraphPad Prism 8.0.2 software (GraphPad Software Inc., San Diego, CA, USA) and are presented as the mean ± standard deviation. Significant differences were identified using a one-way analysis of variance (*P < 0.05, **P < 0.01, ***P < 0.001, ***P < 0.0001).

## Results

### Characterization of CD146^+^ TMSC-NVs derived from TMSCs

**Fig. 1.**
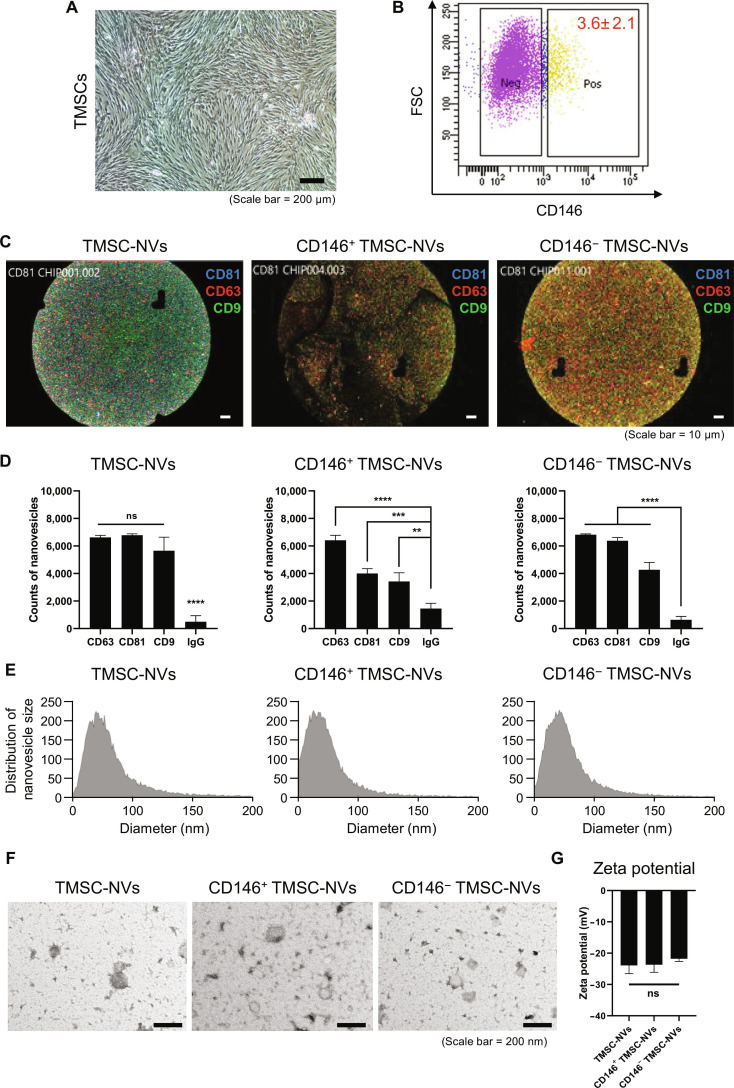
Characterization of Cluster of Differentiation 146 (CD146)-positive nanovesicles derived from tonsil mesenchymal stem cells (CD146^+^ TMSC-NVs). (A) Fibroblast-like morphology of TMSCs (scale bar = 200 μm). (B) Flow cytometry to confirm the population of CD146^+^ TMSCs (*x*-axis: phycoerythrin–CD146; *y*-axis: forward scatter channel [FSC]). (C) Fluorescence imaging using the ExoView R100 platform to confirm the expression of CD63, CD81, and CD9 in CD146^+^ TMSC-NVs (scale bar = 10 μm). (D) The counts of CD146^+^ TMSC-NVs on anti-CD63, anti-CD81, anti-CD9-coated, or immunoglobulin G (IgG) control spots. (E) Size distribution of the NVs. (F) Transmission electron microscopy imaging to confirm the spherical morphology of CD146^+^ TMSC-NVs (scale bar = 200 nm). (G) Zeta potential of NVs. Statistical significance is shown as the mean ± standard deviation (**P < 0.01, ***P < 0.001, ****P < 0.0001; ns, not significant).

The obtained TMSCs were confirmed to have a normal fibroblastic morphology, and flow cytometry revealed the population of CD146^+^ TMSCs to be 3.6% (Fig. 1A and B). To characterize the TMSC-NVs, the expression of surface markers and size distribution were analyzed using the ExoView R100 platform. The TMSC-NVs were captured on CD63, CD81, and CD9 antibody-coated spots, and fluorescence was visualized (Fig. 1C). The number of captured TMSC-NVs was quantified, and the size distribution was measured (Fig. 1D and E). All the NVs expressed CD81, CD63, and CD9, and the mean size distributions were 83 nm for the TMSC-NVs, 76 nm for the CD146^+^ TMSC-NVs, and 80 nm for the CD146^−^ TMSC-NVs. Transmission electron microscopy imaging demonstrated that all the TMSC-NVs displayed a typical spherical phospholipid bilayer (Fig. 1F). In addition, zeta-potential analysis demonstrated that the surface charge of TMSC-NVs was −23.91 ± 2.14 mV, that of CD146^+^ TMSC-NVs was −23.67 ± 2.00 mV, and that of CD146^−^ TMSC-NVs was −21.77 ± 0.69 mV, emphasizing the structural stability of all NVs (Fig. 1G). These results demonstrate that all the TMSC-NVs have similar characteristics to those of exosomes.

### In vitro analysis of CD146^+^ TMSC-NVs in a passage-associated cellular senescence model

**Fig. 2.**
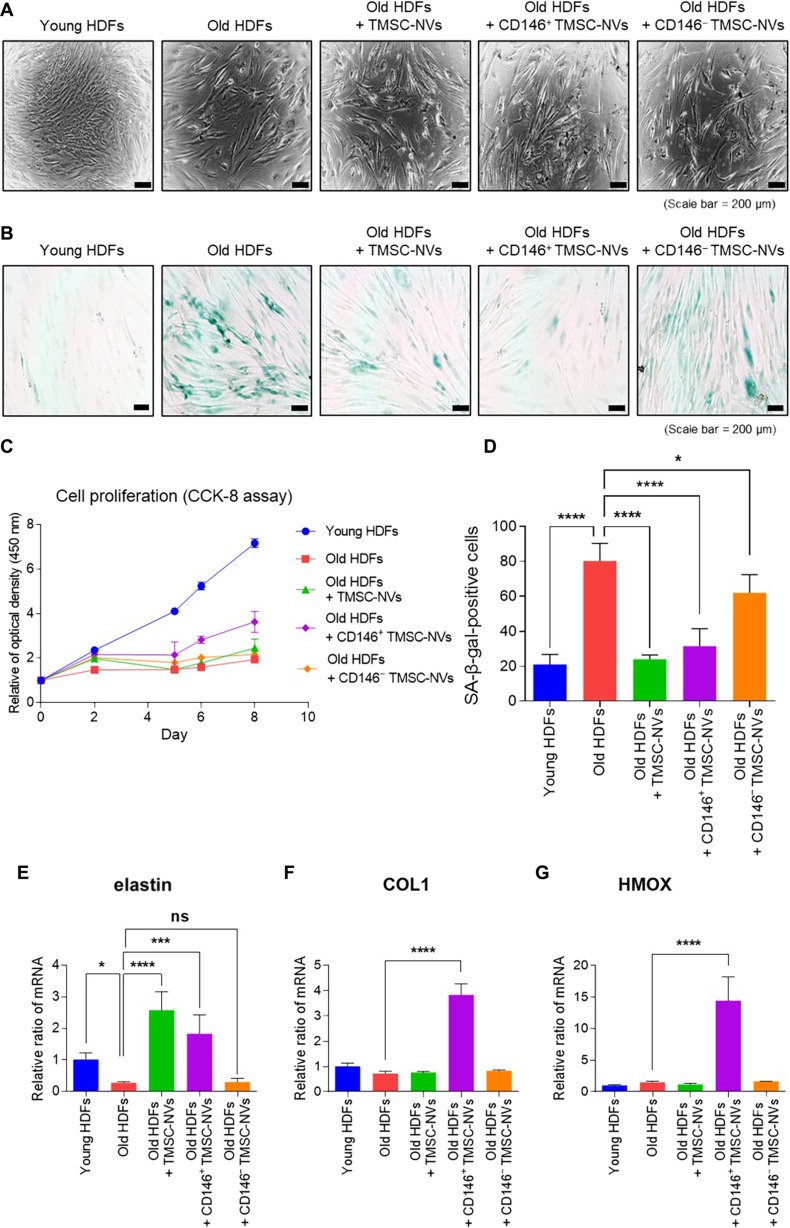
In vitro analysis of CD146^+^ TMSC-NVs in a passage-associated cellular senescence model. (A) Morphological changes of human dermal fibroblasts (HDFs) after treatment with CD146^+^ TMSC-NVs (scale bar = 200 μm). (B) Senescence-associated β-galactosidase (SA-β-gal) assay after treatment with CD146^+^ TMSC-NVs (scale bar = 200 μm). (C) Cell proliferation test using Cell Counting Kit-8 (CCK-8) assay during treatment with CD146^+^ TMSC-NVs. (D) Quantitative data following colorization with the SA-β-gal-positive cells. (E to G) Quantitative data of the polymerase chain reaction for the mRNA expression of elastin, COL1, and HMOX1. Statistical significance is shown as the mean ± standard deviation (*P < 0.05, ***P < 0.001, ****P < 0.0001; ns, not significant).

The CD146^+^ TMSC-NVs were used to treat a passage-associated cellular senescence model to investigate their anti-senescence effects on cellular senescence. Young HDFs exhibited densely packed cultures with small, elongated cell bodies, whereas old HDFs displayed morphological alterations characterized by increased cell size and a flattened shape (Fig. 2A). Treatment with CD146^+^ TMSC-NVs resulted in a denser distribution and the restoration of a smaller, elongated cellular morphology. Cell proliferation was assessed using the Cell Counting Kit-8 assay (Fig. 2C). Treatment with CD146^+^ TMSC-NVs significantly increased the cell proliferation of old HDFs by 187%, whereas treatment with CD146^−^ TMSC-NVs did not. The anti-senescence effects of the CD146^+^ TMSC-NVs were confirmed using the SA-β-gal assay (Fig. 2B). Old HDFs exhibited higher SA-β-gal activity compared to the young HDFs. Both the CD146^+^ TMSC-NVs and TMSC-NVs significantly reduced SA-β-gal activity in the old HDFs. Quantitative data demonstrated a 381% increase in the population of SA-β-gal-positive cells in the old HDFs compared to that in the young HDFs (Fig. 2D). However, treatment with CD146^+^ TMSC-NVs or TMSC-NVs significantly reduced the population of SA-β-gal-positive cells in old HDFs by 70% and 61%, respectively. To evaluate molecular alterations, the expression of ECM components and anti-oxidant genes was evaluated using qPCR. Treatment with CD146^+^ TMSC-NVs significantly increased the expression of elastin and COL1 by 665% and 530%, respectively, compared to that in the old HDFs (Fig. 2E and F). Additionally, the expression of heme oxygenase 1 (HMOX1), an anti-oxidant gene, was increased by 971% following treatment with CD146^+^ TMSC-NVs (Fig. 2G). These findings suggest that CD146^+^ TMSC-NVs effectively mitigate cellular senescence in HDFs.

### Ex vivo analysis of CD146^+^ TMSC-NVs for the dermis in a UVB-induced photoaging model

**Fig. 3.**
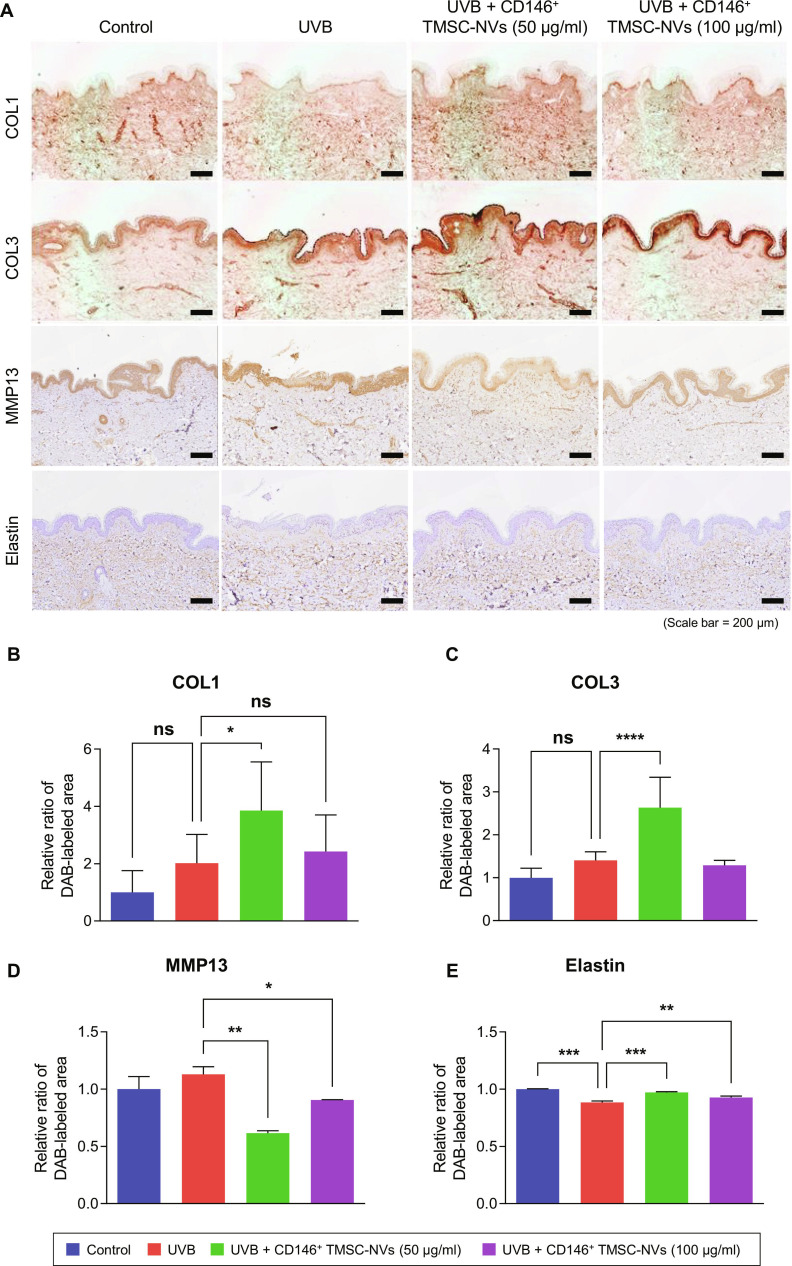
Ex vivo analysis of the dermis in an ultraviolet B (UVB)-induced photoaging model following treatment with CD146^+^ TMSC-NVs. (A) Immunohistochemistry staining to confirm the expression of type I collagen (COL1), type III collagen (COL3), elastin, and matrix metalloproteinase 13 (MMP13) in a UVB-induced photoaging model following treatment with CD146^+^ TMSC-NVs (scale bar = 200 μm). (B to E) Quantitative data of the immunohistochemistry staining in the dermis of the UVB-induced photoaging model following treatment with CD146^+^ TMSC-NVs. Statistical significance is shown as the mean ± standard deviation (*P < 0.05, **P < 0.01, ***P < 0.001, ****P < 0.0001; ns, not significant).

CD146^+^ TMSC-NVs were applied to an ex vivo model of photoaging induced by UVB radiation, based on the results of the passage-associated cellular senescence model. Normal human skin was subjected to UVB irradiation, followed by treatment with 50 or 100 μg/ml of CD146^+^ TMSC-NVs. Immunohistochemistry staining was performed to assess the expression of COL1, COL3, elastin, and MMP13 in the dermis. Treatment with 50 μg/ml of CD146^+^ TMSC-NVs increased the expression of COL1, COL3, and elastin compared to that in the UVB-induced photoaging model and decreased the expression of MMP13 (Fig. 3A). Quantitative data revealed that the expression of COL1, COL3, and elastin were significantly increased by 91%, 87%, and 10%, respectively, following treatment with 50 μg/ml of CD146^+^ TMSC-NVs compared to that in the UVB-induced photoaging model (Fig. 3B to E). In contrast, the expression of MMP13 decreased by 45% compared with that in the UVB-induced photoaging model. Interestingly, treatment with 100 μg/ml of CD146^+^ TMSC-NVs did not regulate the expression of COL1 and COL3 in the UVB-induced photoaging model but increased the expression of elastin by 5% and decreased the expression of MMP13 by 20%. These results indicate that CD146^+^ TMSC-NVs have ECM remodeling properties and suppress MMP activity in the skin in a dose-dependent manner.

### Ex vivo analysis of CD146^+^ TMSC-NVs for the epidermis in a UVB-induced photoaging model

**Fig. 4.**
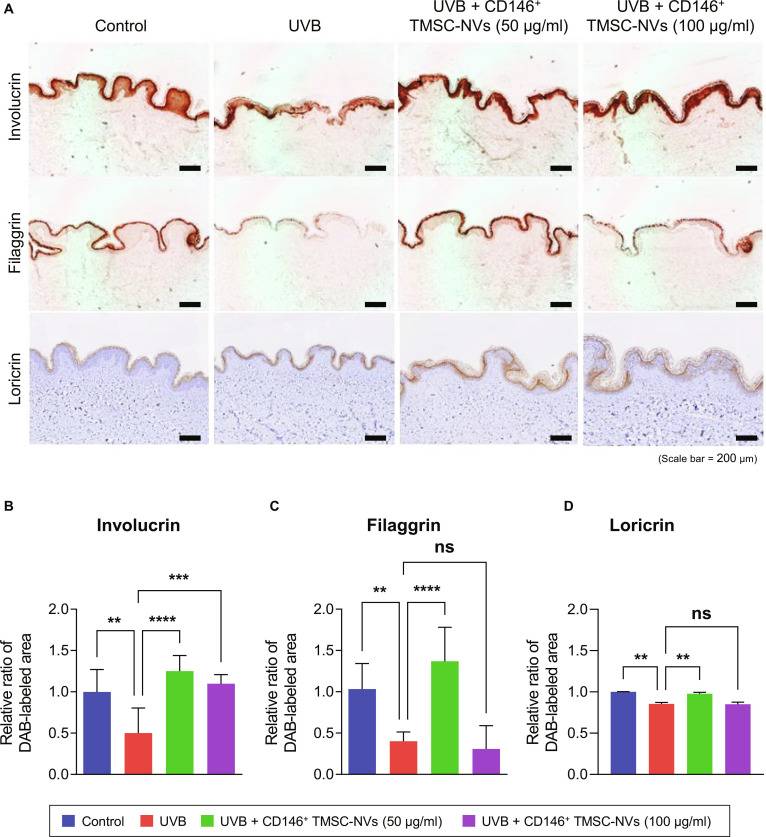
Ex vivo analysis of the epidermis in a UVB-induced photoaging human skin model following treatment with CD146^+^ TMSC-NVs. (A) Immunohistochemistry staining to confirm the expression of involucrin, filaggrin, and loricrin in the UVB-induced photoaging model following treatment with CD146^+^ TMSC-NVs (scale bar = 200 μm). (B to D) Quantitative data of the immunohistochemistry staining in the epidermis of the UVB-induced photoaging model following treatment with CD146^+^ TMSC-NVs. Statistical significance is shown as the mean ± standard deviation (**P < 0.01, ***P < 0.001, ****P < 0.0001; ns, not significant).

Following the ex vivo analysis of the dermis in the UVB-induced photoaging model, immunohistochemistry staining was conducted to assess the expression of involucrin, filaggrin, and loricrin in the epidermis (Fig. 4A). Visual inspection revealed the decreased expression of involucrin, filaggrin, and loricrin in the epidermal layer of the UVB-induced photoaging model compared to that in the control group. In contrast, treatment with 50 μg/ml of CD146^+^ TMSC-NVs increased the expression of involucrin, filaggrin, and loricrin compared to that in the UVB-induced photoaging model. Quantitative data showed that treatment with 50 μg/ml of CD146^+^ TMSC-NVs significantly increased the expression of involucrin, filaggrin, and loricrin by 150%, 240%, and 14%, respectively, compared to that in the UVB-induced photoaging model (Fig. 4B to D). Similar to the observations in the dermis, the restoration of skin barrier was not observed by treatment with 100 μg/ml of CD146^+^ TMSC-NVs. Although the expression of involucrin was significantly increased by 210% through treatment with 100 μg/ml of CD146^+^ TMSC-NVs, the expression of filaggrin was significantly decreased by 24% and significant differences in loricrin were not observed. These results indicate that CD146^+^ TMSC-NVs have ECM remodeling properties and restoration of skin barrier in a dose-dependent manner.

### Cellular uptake analysis

Although the moderate concentration of CD146^+^ TMSC-NVs showed the ECM remodeling properties, suppression of MMP activity and restoration of skin barrier, high concentration of CD146^+^ TMSC-NVs led to a paradoxical collapse of skin barrier function. To determine the optimal working concentration of CD146^+^ TMSC-NVs, cellular internalization efficiency and dose-dependent cytocompatibility of CD146^+^ TMSC-NVs were assessed (Fig. S1). Fluorescence staining using PKH-labeled CD146^+^ TMSC-NVs confirmed successful intracellular internalization, with red fluorescence signals predominantly localized within the cytoplasm (Fig. S1A). Quantitative data of the MFI showed a significant increase by treatment of PKH-labeled CD146^+^ TMSC-NVs. However, no significant difference of MFI was observed between 50 and 100 μg/ml (Fig. S1B). Similar to the observations of fluorescence staining, no significant difference of cell cytotoxicity was observed (Fig. S1C). These results indicate that the cellular uptake mechanism reached saturation at a concentration of 50 μg/ml.

### In vivo analysis of CD146^+^ TMSC-NVs for the skin in an intrinsic aging mice model

**Fig. 5.**
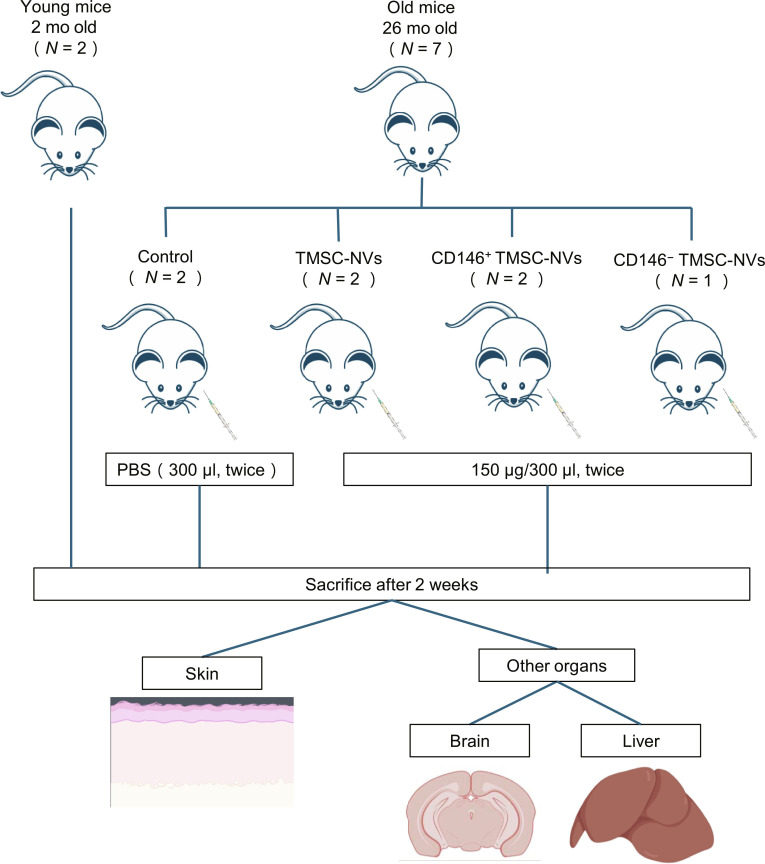
Schematic of the in vivo analysis of CD146^+^ TMSC-NVs using an intrinsic aging mice model. PBS, phosphate-buffered saline.

**Fig. 6.**
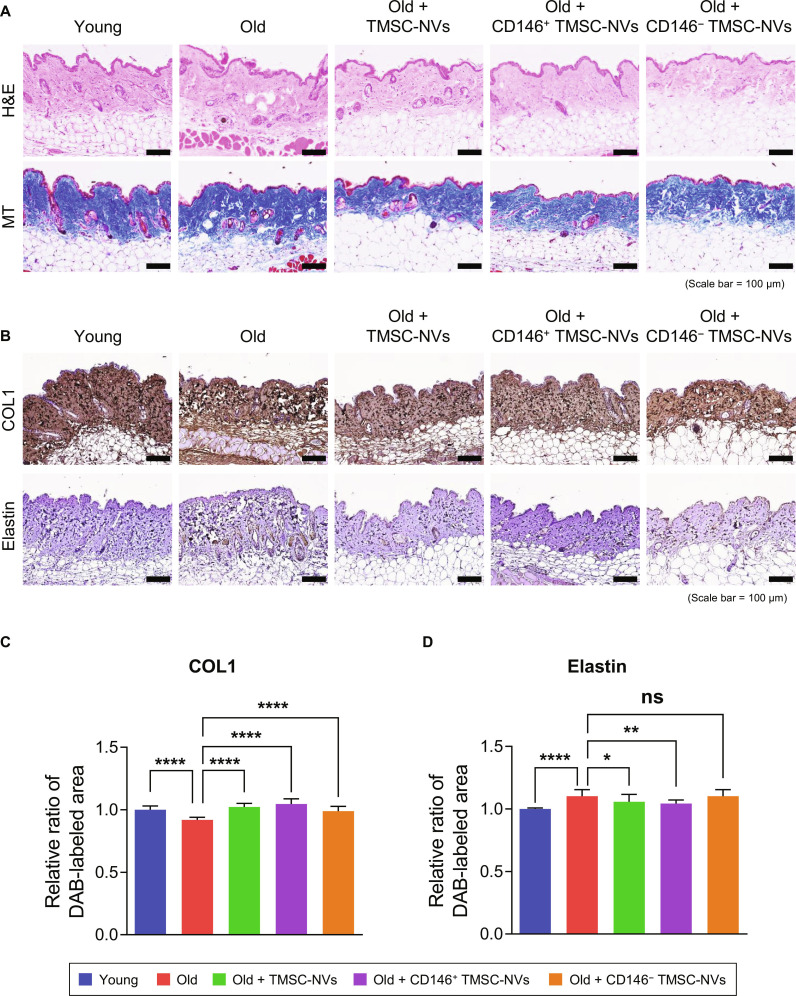
In vivo analysis of the skin in the intrinsic aging mice model after treatment with CD146^+^ TMSC-NVs. (A) Hematoxylin and eosin (H&E) and Masson’s trichrome (MT) staining (scale bar = 100 μm). (B) Immunohistochemistry staining to confirm the expression of COL1 and elastin in the intrinsic aging model following treatment with CD146^+^ TMSC-NVs (scale bar = 100 μm). (C and D) Quantitative data from the immunohistochemistry staining in the skin of the intrinsic aging mice following treatment with CD146^+^ TMSC-NVs. Statistical significance is shown as the mean ± standard deviation (*P < 0.05, **P < 0.01, ****P < 0.0001; ns, not significant).

Animal experiments were conducted according to the scheme summarized in Fig. 5, based on the anti-senescence effects of the CD146^+^ TMSC-NVs. After the completion of the treatment, skin, brain, and liver tissues were surgically obtained. A comprehensive comparison of expression levels was conducted, including a comparison with young mice. Hematoxylin and eosin and Masson’s trichrome staining of the skin revealed that the epidermal layer of the old mice was thinner (Fig. 6A). Additional qualitative changes, such as crypt formation from cell layer collapse and epidermal flattening, were observed. Collagen fibrils in the skin of the old mice appeared fragmented and irregularly distributed at the dermal level compared to those in the young mice. Following the administration of the CD146^+^ TMSC-NVs, the collagen fibrils in the skin of the old mice were densely packed and well organized, whereas administration of CD146^−^ TMSC-NVs did not cause notable structural changes. Immunohistochemistry staining was performed to assess the expression of COL1 and elastin in the skin of the old mice (Fig. 6B). Visual inspection revealed that the expression of COL1 in the skin of the old mice was lower than that in the skin of the young mice, whereas the expression of elastin was higher. In contrast, the administration of CD146^+^ TMSC-NVs increased the expression of COL1 and decreased the expression of elastin compared to that in the old mice. Quantitative data showed that the relative expression of COL1 decreased by 8% in the skin of the old mice compared to that in the young mice, whereas the expression of elastin increased by 10% (Fig. 6C and D). The administration of TMSC-NVs, CD146^+^ TMSC-NVs, and CD146^−^ TMSC-NVs significantly increased the expression of COL1 by 11%, 14%, and 7% compared to that in the skin of the old mice, respectively. Only the administration of CD146^+^ TMSC-NVs decreased elastin expression by 5%. These results indicate that CD146^+^ TMSC-NVs have anti-senescence effects in the skin.

### In vivo analysis of CD146^+^ TMSC-NVs for the brain in an intrinsic aging mice model

**Fig. 7.**
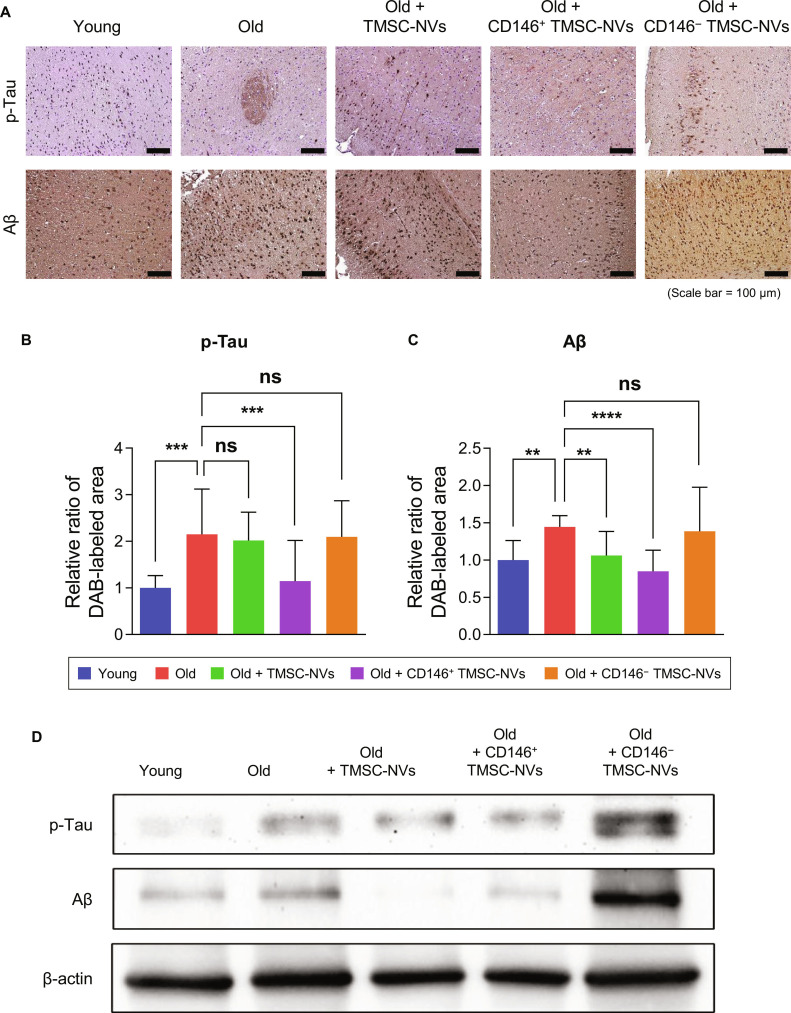
In vivo analysis of the brain in the intrinsic aging mice model after treatment with CD146^+^ TMSC-NVs. (A) Immunohistochemistry staining to confirm the expression of phospho-Tau (p-Tau) and amyloid beta (Aβ) in the intrinsic aging model after treatment with CD146^+^ TMSC-NVs (scale bar = 100 μm). (B and C) Quantitative data from the immunohistochemistry staining. (D) Western blotting of p-Tau, Aβ, and β-actin from the brains of the intrinsic aging mice model after treatment with CD146^+^ TMSC-NVs. Statistical significance is shown as the mean ± standard deviation (**P < 0.01, ***P < 0.001, ****P < 0.0001; ns, not significant).

The expression levels of p-Tau and Aβ, markers of Alzheimer’s disease, were assessed using immunohistochemistry staining in the brains from old mice. Visual inspection revealed that the expression of p-Tau was higher in the brains of old mice than in those of young mice (Fig. 7A). However, the administration of CD146^+^ TMSC-NVs reduced the expression of p-Tau in the brains of old mice, resembling the levels observed in young mice. Quantitative data showed that the expression of p-Tau and Aβ in the brains from the old mice was significantly increased by 215% and 145%, respectively, compared to that in the young mice, which was reduced following the administration of CD146^+^ TMSC-NVs by 47% and 41%, respectively (Fig. 7B and C). Western blotting was performed to investigate the expression of p-Tau and Aβ in the brain (Fig. 7D). The administration of CD146^+^ TMSC-NVs reduced the expression of p-Tau and Aβ compared to that in the old mice, whereas CD146^−^ TMSC-NVs increased the expression. These results indicate that CD146^+^ TMSC-NVs have anti-senescence effects in the brain.

### In vivo analysis of CD146^+^ TMSC-NVs for the liver in an intrinsic aging mice model

**Fig. 8.**
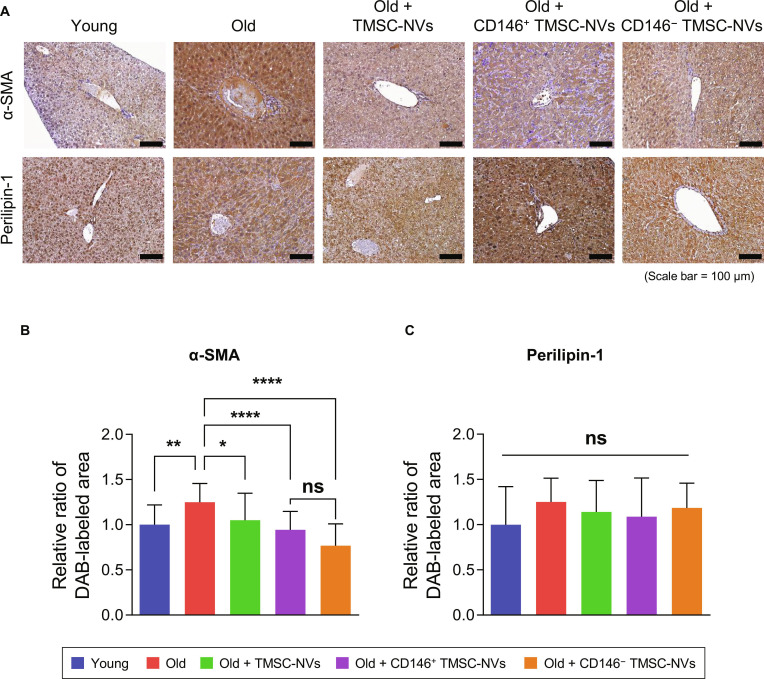
In vivo analysis of the liver in the intrinsic aging mice model after treatment with CD146^+^ TMSC-NVs. (A) Immunohistochemistry staining to confirm the expression of alpha-smooth muscle actin (α-SMA) and perilipin-1 in the liver from the intrinsic aging model after treatment with CD146^+^ TMSC-NVs (scale bar = 100 μm). (B and C) Quantitative data from the immunohistochemistry staining. Statistical significance is shown as the mean ± standard deviation (*P < 0.05, **P < 0.01, ****P < 0.0001; ns, not significant).

The expression of α-SMA, a marker of liver fibrosis, and perilipin-1, a marker of hepatic fibrosis and steatosis, was assessed in the liver using immunohistochemistry staining (Fig. 8A). The relative expression of α-SMA was significantly increased in the liver of the old mice compared to that in the young mice. Quantitative data revealed that the administration of TMSC-NVs, CD146^+^ TMSC-NVs, and CD146^−^ TMSC-NVs reduced the expression of α-SMA by 16%, 24%, and 38%, respectively, compared to that in the old mice, but no significant differences were observed between the administration of CD146^+^ TMSC-NVs and CD146^−^ TMSC-NVs (Fig. 8B and C). However, no significant differences were observed in the expression of perilipin-1 among the mice. These results indicate that CD146^+^ TMSC-NVs have anti-senescence effects on liver fibrosis.

### Microarray analysis of CD146^+^ TMSC-NVs

Microarray analysis was conducted using the Affymetrix WT Expression Microarray to assess the molecular mechanisms underlying the anti-senescence effects of the CD146^+^ TMSC-NVs (Fig. S2). A total of 21,488 probes were used to analyze the differential expression among the HDFs. The expression of the whole transcriptome and the similarity of each group were determined using a hierarchical clustering heatmap (Fig. S2A). The results revealed that the gene expression patterns were most similarly regulated following treatment with TMSC-NVs and CD146^−^ TMSC-NVs. Additionally, gene expression with a fold change greater than the cutoff value (fold change >2 or <−2, *P* < 0.05) was classified using a scatter plot of DEGs (Fig. S2B). In total, 1,004 genes were found to be increased, and 921 genes were decreased in young HDFs compared to those in old HDFs. The expression of 1,106 genes was increased following treatment with CD146^+^ TMSC-NVs, whereas the expression of 1,121 genes was decreased compared to that in old HDFs. Treatment with TMSC-NVs resulted in the higher expression of 778 genes and lower expression of 655 genes. The expression of 803 genes was increased and that of 790 genes was decreased following treatment with CD146^−^ TMSC-NVs compared to that in the old HDFs. Next, Gene Ontology enrichment analysis was performed using gprofiler to analyze the functional changes after treatment with CD146^+^ TMSC-NVs, and the results were classified into 3 categories: biological processes, molecular functions, and cellular components (Fig. S2C). A functional analysis of the biological processes enriched following treatment with CD146^+^ TMSC-NVs highlighted the developmental process, anatomical structure development, regulation of cellular process, multicellular organismal process and development, response to stimulus, and system-development-related genes. These results were similar to the comparison between the young and old HDFs. In addition, a functional analysis of the cellular components showed that treatment with CD146^+^ TMSC-NVs significantly enriched the cytoplasm, cell periphery, extracellular region, vesicle, and extracellular-space-related genes. Treatment with CD146^+^ TMSC-NVs significantly enriched ECM structural constituents, integrin binding, and cytokine-activity-related genes. To further assess the senescence-associated molecular mechanisms of the CD146^+^ TMSC-NVs, DEGs were analyzed using KEGG pathway analysis (Fig. S3). The KEGG pathway analysis of the cell cycle and the apoptosis showed that treatment with CD146^+^ TMSC-NVs regulated the p300-related p53 signaling pathway (Fig. S3A and B). The KEGG pathway analysis of the p53 signaling demonstrated that treatment with CD146^+^ TMSC-NVs decreased the mRNA expression of pro-apoptotic genes such as p53, Fas, Growth arrest and DNA damage-inducible 45 (GADD45), and Sestrins, while increasing the mRNA expression of Bcl2, which demonstrated the modulation of the p53 pathway (Fig. S3C). Interestingly, the mRNA expression of Ataxia Telangiectasia Mutated (ATM) and Ataxia Telangiectasia and Rad3 Related (ATR) subfamilies increased, and that of MDM2-like p53-binding protein (MDM-X), a negative regulator of p53 was decreased. These results indicate that CD146^+^ TMSC-NVs modulate apoptosis through the p53 signaling pathway.

### Anti-senescence properties of CD146^+^ TMSC-NVs via modulation of the p53 signaling pathway

**Fig. 9.**
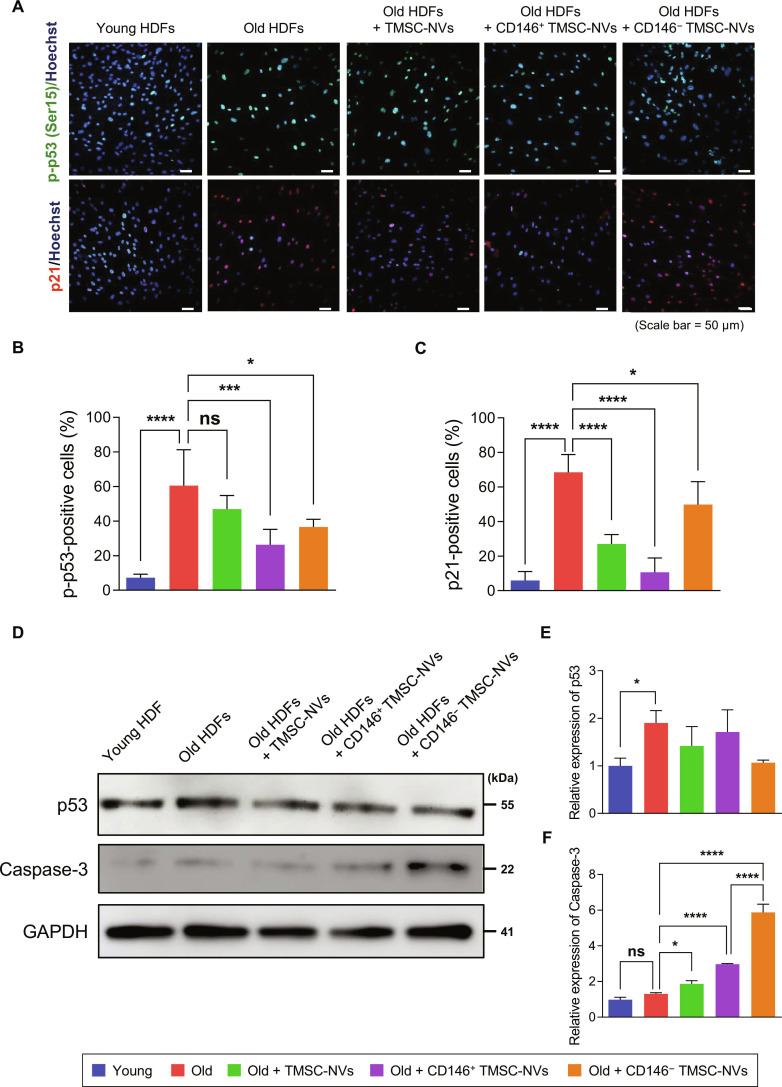
Anti-senescence properties of CD146^+^ TMSC-NVs via modulation of the p53 signaling pathway. (A) Immunofluorescence image to confirm the expression of p-p53 and p21 after treatment with CD146^+^ TMSC-NVs. p-p53 (green, top row) and p21 (red, bottom row) were co-stained with Hoechst 33342 (blue) to visualize the nuclei (scale bars = 50 μm). (B) Quantitative data of p-p53-positive cells. (C) Quantitative data of p21-positive cells. (D) Western blotting of p53 and caspase-3. Glyceraldehyde 3-phosphate dehydrogenase (GAPDH) was used as an endogenous control. (E) Quantitative data of western blotting of p53. (F) Quantitative data of western blotting of caspase-3. Statistical significance is shown as the mean ± standard deviation (*P < 0.05, ***P < 0.001, ****P < 0.0001; ns, not significant).

To determine whether the modulation of p53 signaling pathway was reflected on the protein expression, the phosphorylation of p53 at Ser15 (p-p53), a key upstream sensor for cellular stress. and the expression of its downstream target, p21, were assessed by immunofluorescence staining (Fig. 9A). As a result, it is revealed that the expression of p-p53 and p21 in old HDFs was significantly decreased by treatment of CD146^+^ TMSC-NVs. Quantitative data showed that the population of p-p53-positive cells was 60% in old HDFs, whereas the population of p-p53-positive cells decreased to 26% by treatment of CD146^+^ TMSC-NVs (Fig. 9B). The population of p21-positive cells was 68% in old HDFs, whereas the population of p21-positive cells decreased to 10% by treatment of CD146^+^ TMSC-NVs (Fig. 9C). In addition, the expression of total p53 was assessed by western blotting, and quantitative data showed that there are no significant differences in the expression of total p53, except in old HDFs (Fig. 9D and E). These results indicate that CD146^+^ TMSC-NVs decreased the cellular senescence by modulation of the p53–p21 signaling axis without altering basal p53 homeostasis. Interestingly, it was revealed that the expression caspase-3 was highly increased by treatment with CD146^+^ TMSC-NVs and CD146^−^ TMSC-NVs, although the expression of cleaved caspase-3, the activated protein, was not detected (data not shown), indicating that the treatment with those NVs did not trigger the terminal apoptotic cascade (Fig. 9D and F).

### miRNA profiling of CD146^+^ TMSC-NVs

**Fig. 10.**
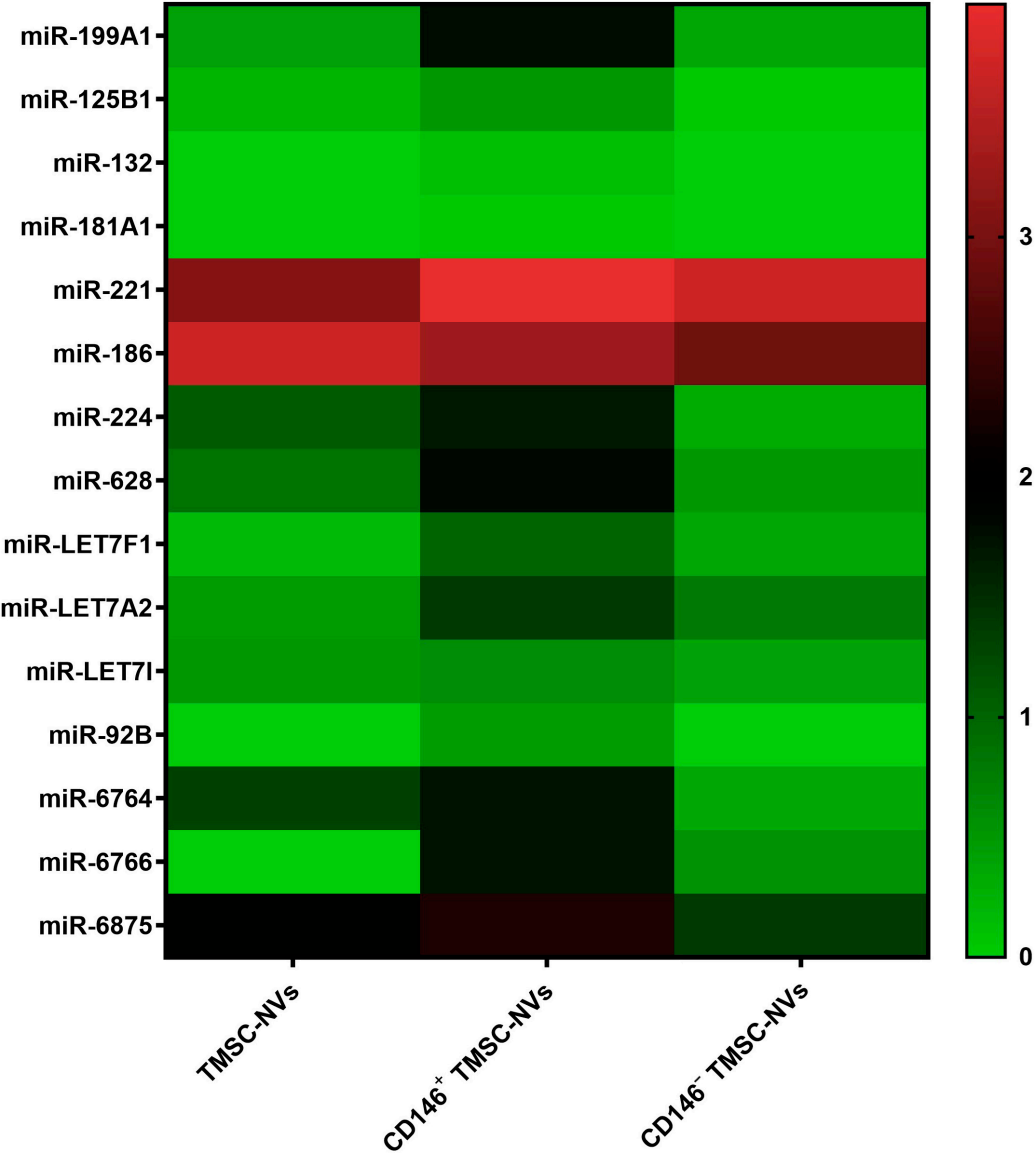
miRNA profiling. Heatmap showing selected miRNAs enriched in CD146^+^ TMSC-NVs. miRNAs were categorized based on their functional relevance to extracellular matrix remodeling, oxidative stress, and cellular senescence, along with highly enriched miRNAs representing CD146^+^ TMSC-NVs. Expression values were normalized as log2(TPM + 1).

To determine the components within CD146^+^ TMSC-NVs, miRNA profiling was conducted using RNA sequencing (Fig. 10). As a result, it was demonstrated that CD146^+^ TMSC-NVs have selectively enriched miRNAs associated with anti-senescence. Notably, miRNAs implicated in ECM remodeling and cellular homeostasis, including miR-199A1, miR-181A1, miR-125b, and miR-132, showed relatively higher expression in CD146^+^ TMSC-NVs compared to other groups. In addition, miRNAs related to cell proliferation and senescence regulation, such as miR-221, miR-186, and miR-224, were also up-regulated. Interestingly, a subset of highly abundant but less-characterized miRNAs, including miR-6875, miR-6764, and miR-6766, exhibited markedly elevated expression in CD146^+^ TMSC-NVs, suggesting the presence of a distinct molecular signature. Collectively, these results indicate that CD146^+^ TMSC-NVs harbor a coordinated miRNA profile associated with ECM remodeling, oxidative stress regulation, and suppression of cellular senescence.

## Discussion

A previous study revealed that TMSC-NVs improve cell proliferation and reduce senescence in old HDFs [15]. TMSC-NVs may therefore serve as an alternative to exosomes for skin rejuvenation. However, the mechanisms by which TMSC-NVs regulate cellular senescence have been unclear. This study was therefore aimed at determining the anti-senescence effects of TMSC-NVs and their ability to regulate cellular senescence.

In the present study, TMSCs were separated into CD146^+^ and CD146^−^ TMSCs using magnetic-activated cell sorting. TMSC-NVs, CD146^+^ TMSC-NVs, and CD146- TMSC-NVs were manufactured via serial extrusion and had similar characteristics to exosomes (Fig. 1C to G). Treatment with CD146^+^ TMSC-NVs significantly increased cell proliferation and reduced SA-β-gal activity in a passage-associated cellular senescence in vitro model, whereas CD146^−^ TMSC-NVs did not exhibit this effect (Fig. 2A to D). A qPCR analysis revealed that the mRNA expression of ECM and anti-oxidant genes was increased following treatment with CD146^+^ TMSC-NVs (Fig. 2E to G). These results indicated that the CD146^+^ TMSC-NVs decreased cellular senescence and increased cell proliferation, anti-oxidant gene expression, and ECM remodeling. Therefore, CD146^+^ TMSC-NVs reduced cellular senescence and demonstrated the anti-senescence effects.

In an ex vivo analysis, treatment with CD146^+^ TMSC-NVs significantly increased the expression of COL1, COL3, and elastin and decreased that of MMP13 in the dermis compared to that in the UVB-induced photoaging model (Fig. 3). In addition, treatment with CD146^+^ TMSC-NVs also significantly increased the expression of involucrin, filaggrin, and loricrin (Fig. 4). These proteins are known to play a crucial role in maintaining skin barrier function, especially in response to UV radiation, in the epidermal layer [25]. UV radiation disrupts the expression of these proteins, thereby compromising the protective barrier and rendering the skin more susceptible to dehydration, damage, and photoaging [26]. These results indicate that CD146^+^ TMSC-NVs promotes restoration of skin barrier as well as ECM remodeling by suppression of MMP expression, emphasizing their therapeutic potential for skin rejuvenation.

Although CD146^+^ TMSC-NVs exhibit significant therapeutic potential, this effect was not observed at higher concentrations. To interpret these results, cellular internalization efficiency and dose-dependent cytocompatibility of CD146^+^ TMSC-NVs were assessed (Fig. S1). As a result, the significant differences of cytotoxicity and cellular uptake were not observed, indicating that CD146^+^ TMSC-NVs reached a saturation point at concentration of 50 μg/ml. Furthermore, high concentrations of NVs have been reported to trigger diverse side effects such as metabolic stress, unintended immune responses, or nonspecific cellular changes [27]. These results may contribute to optimizing concentration of NVs, providing maximal anti-senescent efficacy while avoiding the unexpected risks associated with high-dose NV exposure.

An in vivo analysis of the intrinsic aging mice model was conducted to evaluate the anti-senescence effects of the CD146^+^ TMSC-NVs. All the NVs were administered to the intrinsic aging mice model through intraperitoneal administration, which involves delivering a drug directly into the peritoneal cavity [28]. Administration of CD146^+^ TMSC-NVs improved collagen fibril density and organization, which exhibited fragmented and irregular collagen distribution (Fig. 6A). Notably, the expression of COL1 in the dermis of the old mice was lower than that in the dermis of the young mice, whereas elastin expression was higher (Fig. 6B to D). The increase of elastin has been reported in previous rodent model studies [29]. These results are expected to be an aging-related elastosis-like phenotype resulting from the accumulation of degraded elastin rather than the increase of functional elastin. In the present study, administration of CD146^+^ TMSC-NVs increased the expression of COL1 and reduced the accumulation of elastin. Based on the in vitro and ex vivo analysis, these results suggest that CD146^+^ TMSC-NVs do not simply increase ECM components but restore ECM homeostasis by functional restoration of cellular senescence. Collectively, CD146^+^ TMSC-NVs may improve dermal integrity through coordinated regulation of cellular function and ECM remodeling.

Intraperitoneal administration is expected to predominantly affect various organs, including the liver and brain, as well as the brain–blood barrier (BBB) [30]. The BBB is a highly selective, semipermeable barrier that protects the brain from potentially harmful substances in the bloodstream, including most cells, drugs, and larger molecules. However, NVs exhibit a significant advantage over stem cells in terms of crossing the BBB because of their small size and capacity to be internalized by brain endothelial cells [31]. In the present study, the expression of aging-related proteins and the Alzheimer’s disease markers, Aβ and p-Tau, was assessed to demonstrate the anti-senescence effects of the CD146^+^ TMSC-NVs in the brain (Fig. 7). The expression of p-Tau and Aβ was higher in the brains of the old mice than that in the young mice. Aβ and p-Tau have been widely recognized as key contributors to the pathophysiology of Alzheimer’s disease, largely due to their accumulation in hallmark histopathological lesions and increased levels of their soluble forms in the brains of patients with Alzheimer’s disease [32–34]. In the present study, immunohistochemistry staining and western blotting revealed that treatment with CD146^+^ TMSC-NVs significantly reduced the expression of p-Tau and Aβ in the brains of the old mice. These results indicate that CD146^+^ TMSC-NVs may play a role in decreasing the expression of p-Tau and Aβ, demonstrating potential neuroprotective effects. Whether through direct BBB penetration or indirect signaling, the precise delivery route remains to be fully elucidated. Further investigation into the specific molecular pathways and long-term biodistribution will be essential to validate CD146^+^ TMSC-NVs as a novel therapeutic strategy for Alzheimer’s disease.

Numerous studies have reported that MSC-derived extracellular vesicles and exosomes accumulate in liver tissue after intraperitoneal, intravascular, and direct administration [35–37]. Liver tissue was obtained to assess the anti-senescence effects of CD146^+^ TMSC-NVs. The expression of α-SMA and perilipin-1 in the liver was assessed by immunohistochemistry staining (Fig. 8). α-SMA is widely recognized as a reliable biomarker for assessing liver fibrosis, as its levels strongly correlate with fibrosis severity [10]. Similarly, perilipin-1, which is predominantly expressed in mature adipocytes, is mainly observed in large lipid droplets and plays a role in the development of macrovascular steatosis in hepatocytes [38]. In the present study, significant differences in the expression of α-SMA were found between the old and young groups. The CD146^+^ TMSC-NV-treated group demonstrated a significant decrease in α-SMA expression compared to that in the old mice. These results indicate that TMSC-NVs exert antifibrotic effects on liver fibrosis. However, no significant differences were observed in the expression of perilipin-1 among all mice. This suggests that the intrinsic aging model in the present study may be characterized by mild steatosis, where the accumulation of lipid droplet has not yet reached a pathological threshold requiring metabolic intervention. Further investigation is warranted to identify whether alternative disease-modeling strategies or treatment durations yield different outcomes, particularly for the potential therapeutic applications of CD146^+^ TMSC-NVs in fatty liver disease.

This preliminary in vivo analysis was performed on 26-month-old mice, corresponding to a late-life stage characterized by sharply increased mortality [39]. To ensure the statistical power of the study, further investigation will incorporate alternative aging models such as the Senescence-Accelerated Mouse (SAM), D-galactose-administered mice, or moderately aged mice (e.g., 12 mo old), thereby allowing for more statistically robust group sizes.

Microarray analysis was conducted to assess the anti-senescence effects of CD146^+^ TMSC-NVs on the gene expression profiles of HDFs. The results indicated that senescence-related genes were differentially expressed in HDFs treated with CD146^+^ TMSC-NVs compared to those in old HDFs (Fig. S2). In addition, a KEGG pathway analysis demonstrated that CD146^+^ TMSC-NVs modulated the apoptosis pathway (Fig. S3). ATM and ATR, regarded as major players in the DNA damage response, are known to elevate cellular senescence and apoptosis via the phosphorylation of p53 through its signaling pathway [40], and MDM-X is regarded as a negative regulator of p53 by binding to p53 [41]. Interestingly, despite the increase of ATM and ATR expression and the decrease of MDM-X expression in upstream, the mRNA expression of p53 was decreased following treatment with CD146^+^ TMSC-NVs. This apparent paradox can be interpreted as a successful resolution of the senescence-associated signaling cascade. While the increased expression of ATM and ATR suggests active engagement in the DNA damage response or repair pathways, the subsequent decreased the mRNA expression of p53 indicate that the p53-mediated senescence signaling was effectively decreased by CD146^+^ TMSC-NVs. Furthermore, the decrease of population of p-p53 and p21 was observed by treatment with CD146^+^ TMSC-NVs (Fig. 9). These results indicate that CD146^+^ TMSC-NVs decreased the cellular senescence by modulation of the p53–p21 signaling axis without altering basal p53 homeostasis.

However, the expression of caspase-3 was increased by treatment with CD146^+^ TMSC-NVs and CD146^−^ TMSC-NVs, except the expression of cleaved caspase-3 (Fig. 9D and F). Typically, senescent cells acquire resistance to apoptosis to maintain survival [42]. However, exposure to severe cellular stress or senolytic stimuli can disrupt these survival networks, driving them into an apoptotic transition phase. In the present study, CD146^+^ TMSC-NVs effectively attenuated cellular senescence by modulating the p53–p21 axis; notably, neither significant cytotoxicity nor the expression of active cleaved caspase-3 was observed. Collectively, these results are highly consistent with our previous microarray analysis, further strengthening the proposed mechanism that CD146^+^ TMSC-NVs provide a therapeutic potential for reversing cellular senescence.

Additionally, miRNA profiling was conducted to determine the efficient ingredients of CD146^+^ TMSC-NVs for anti-senescence properties (Fig. 10). Several miRNAs enriched in CD146^+^ TMSC-NVs, including miR-199A1, miR-181A1, miR-125b, and miR-132, have been reported to function as bioactive cargos in extracellular vesicles, regulating ECM remodeling, inflammatory responses, and oxidative stress. In particular, exosomal miR-199a and miR-181a are known to modulate ECM turnover and MMP activity [43,44], while miR-125b and miR-132 contribute to anti-inflammatory and anti-oxidant effects [45,46]. Additionally, miR-186, miR-221, and miR-224 have been implicated in promoting cell proliferation and suppressing senescence-related pathways [47–49]. These findings support the notion that the anti-aging effects of CD146^+^ TMSC-NVs are mediated, at least in part, by functionally relevant miRNA cargos. Collectively, these results indicate that CD146^+^ TMSC-NVs harbor a coordinated multi-miRNA signature associated with ECM remodeling, oxidative stress regulation, and cellular rejuvenation, emphasizing the anti-senescence properties for clinical applications. Meanwhile, a subset of highly abundant miRNAs, including miR-6875, miR-6764, and miR-6766, exhibited markedly elevated expression in CD146^+^ TMSC-NVs. To date, these miRNAs have not been functionally characterized in the context of anti-senescence or ECM remodeling. However, their prominent enrichment suggests that they may represent previously unrecognized regulatory components associated with CD146^+^ TMSC-NV-mediated biological effects. Given their high expression levels, further investigation of these miRNAs may contribute to anti-aging processes through mechanisms that are not yet fully understood.

The present study revealed that treatment with CD146^+^ TMSC-NVs decreased cellular senescence by modulation of p53 pathway. In vitro analysis demonstrated that CD146^+^ TMSC-NVs increased cell proliferation and decreased the cellular senescence. Ex vivo analysis using the UVB-induced photoaging model demonstrated the anti-senescence effects of CD146^+^ TMSC-NVs through MMP suppression and ECM remodeling capacity. In vivo analysis using the intrinsic aging mice model revealed that treatment with CD146^+^ TMSC-NVs increased ECM remodeling and reversed aging-related disease. Microarray analysis, immunofluorescence staining, and miRNA profiling demonstrated the anti-senescence mechanism of CD146^+^ TMSC-NVs through modulation of the p53 pathway. These results indicate the significant therapeutic potential of CD146^+^ TMSC-NVs in anti-senescence therapies.

## Data Availability

The data that support the findings of this study are available from the corresponding author upon reasonable request.
