# Supplementary material for: Anti-senescence Effects of Nanovesicles Derived from Cluster of Differentiation-146-Positive Tonsil Mesenchymal Stem Cells via Modulation of the Tumor Protein 53 Pathway
Source: Biomater Res. 2026 May 21;30:0371. doi: 10.34133/bmr.0371 (PMC13191092; doi:10.34133/bmr.0371)
Supplement: Supplementary 1 — Figs. S1 to S3 Table S1 [file bmr.0371.f1.zip › Supplementary Materials.docx]

**Supplementary Materials**

**Fig. S1.** Cellular uptake and cytocompatibility of CD146^+^ TMSC-NVs in HDFs. (A) Representative fluorescence images showing the internalization of PKH-labeled CD146^+^ TMSC-NVs in HDFs. Cells were treated with 0, 50 and 100 μg/mL of CD146^+^ TMSC-NVs for 24 h. Red: PKH-labeled NVs; Green: F-actin (Phalloidin); Blue: Nuclei (Hoechst 33342) (Scale bars = 50 μm). (B) Quantitative data of the Mean Fluorescence Intensity (MFI). (C) Quantitative data of cell viability using the CCK-8 assay. Statistical significance was shown as the mean ± standard (** P < 0.01, *** P < 0.001).

**Fig. S2.** Microarray analysis. The Affymetrix® WT Expression Microarray was utilized to assess the anti-senescence effects of CD146^+^ TMSC-NVs on the gene expression profiles of HDFs. (A) Hierarchical clustering heatmap. (B) Scattering plot of DEGs with a fold change greater than the cut-off value (fold change > 2 or < −2, *P* < 0.05). (C) Gene enrichment and functional annotation analysis using gprofiler.

**Fig. S3.** KEGG pathway analysis though microarray analysis. (A) KEGG pathway analysis of the cell cycle. (B) KEGG pathway analysis of the apoptosis. (C) KEGG pathway analysis of the p53 pathway.

**Table. S1.** Primer pairs used for qPCR.
