# Supplementary material for: Anti-senescence Effects of Nanovesicles Derived from Cluster of Differentiation-146-Positive Tonsil Mesenchymal Stem Cells via Modulation of the Tumor Protein 53 Pathway
Source: Biomater Res. 2026 May 21;30:0371. doi: 10.34133/bmr.0371 (PMC13191092; doi:10.34133/bmr.0371)
Supplement: Supplementary 1 — Figs. S1 to S3 Table S1 [file bmr.0371.f1.zip › Table S1.pdf]

# Supplementary Materials 4

| *Gene Name* | *Gene Bank Number* | *Forward (5'– 3')* | *Reverse (5'– 3')* |
|---|---|---|---|
| ***GAPDH*** | NM_002046.7 | ACA TCG CTC AGA CAC CAT G | TGT AGT TGA GGT CAA TGA AGG G |
| ***COL1*** | NM_000089.4 | ACA AGG CAT TCG TGG CGA TA | ACC ATG GTG ACC AGC GAT AC |
| ***Elastin*** | NM_001081755.3 | CAT TTC TCC CCG AGA TGG CG | CGA GAC CAG CCC CTG GAT AA |
| ***HMOX1*** | NM_002133.3 | AGA CAC CCT AAT GTG GCA GC | ATG GCC GTG TCA ACA AGG AT |

**Table. S1.** Primer pairs used for qPCR.
