# Supplementary figures and images for: Anti-senescence Effects of Nanovesicles Derived from Cluster of Differentiation-146-Positive Tonsil Mesenchymal Stem Cells via Modulation of the Tumor Protein 53 Pathway
Source: Biomater Res. 2026 May 21;30:0371. doi: 10.34133/bmr.0371 (PMC13191092; doi:10.34133/bmr.0371)

# Supplementary Materials 1

**A**

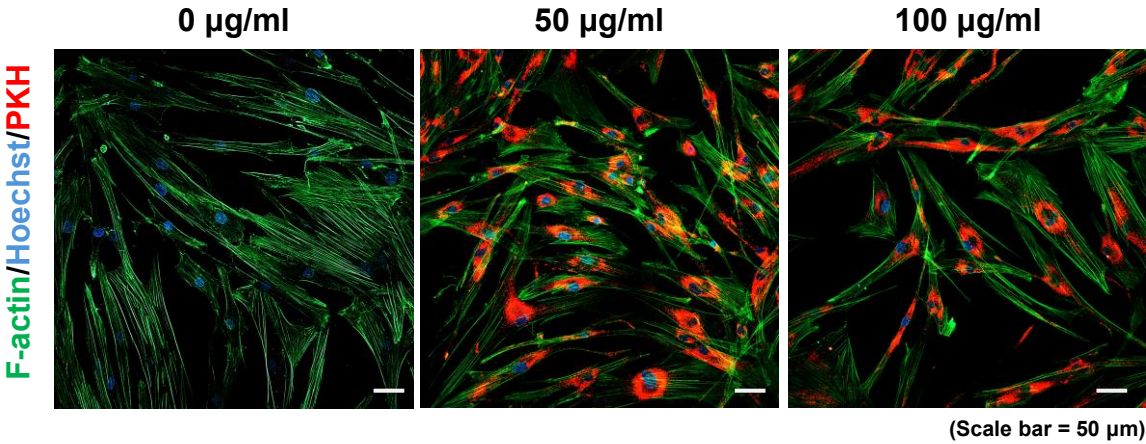

**B**

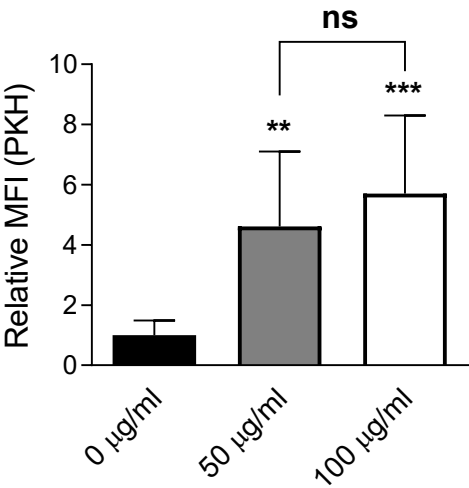

**C**

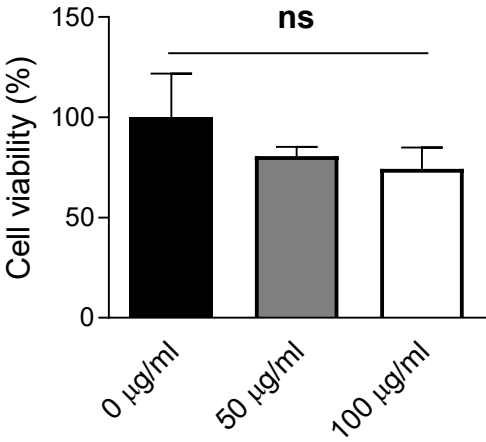

Supplement: Supplementary 1 — Figs. S1 to S3 Table S1 [file bmr.0371.f1.zip › Figure S1.pdf]

# Supplementary Materials 2

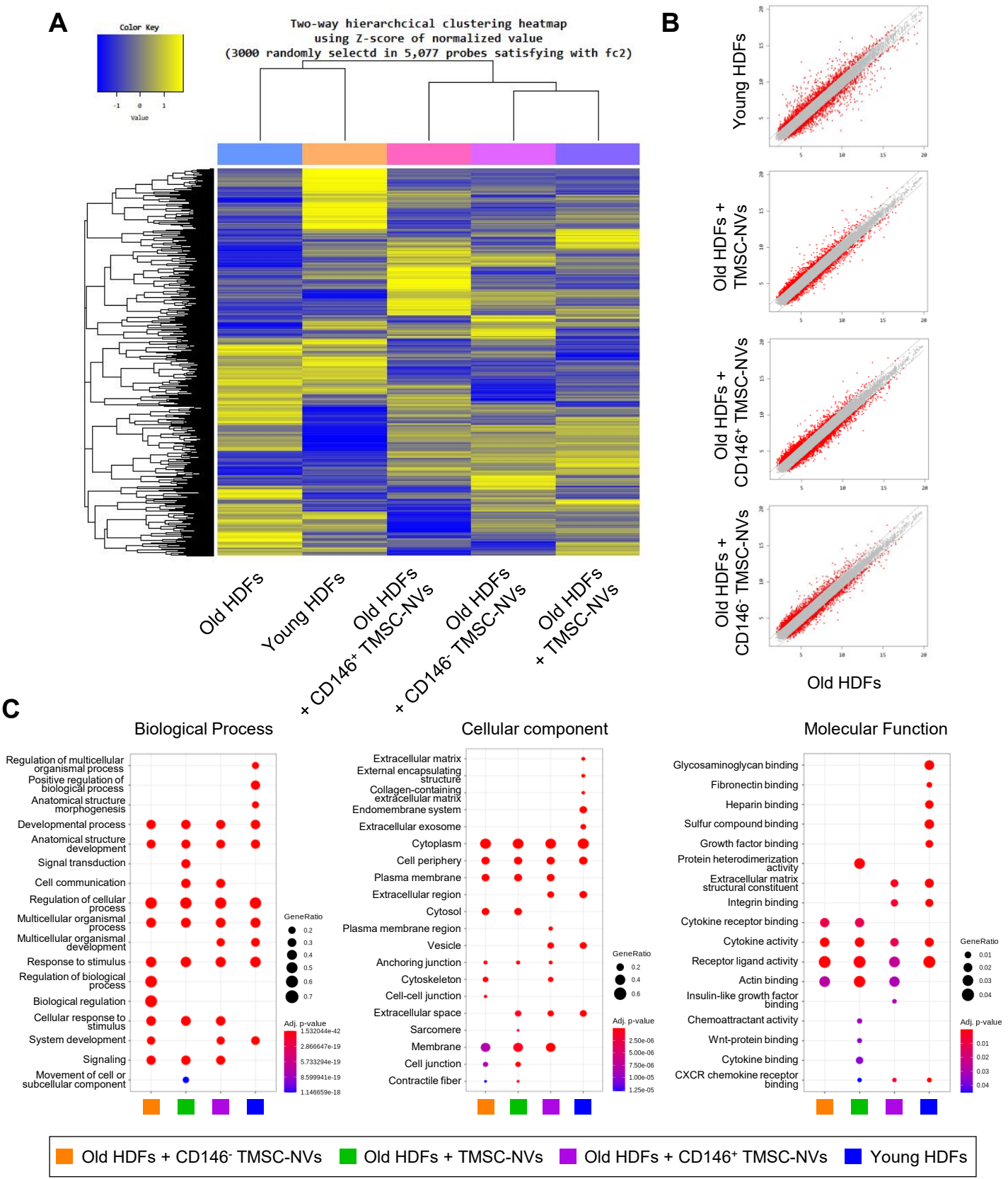

Supplement: Supplementary 1 — Figs. S1 to S3 Table S1 [file bmr.0371.f1.zip › Figure S2.pdf]

**A**

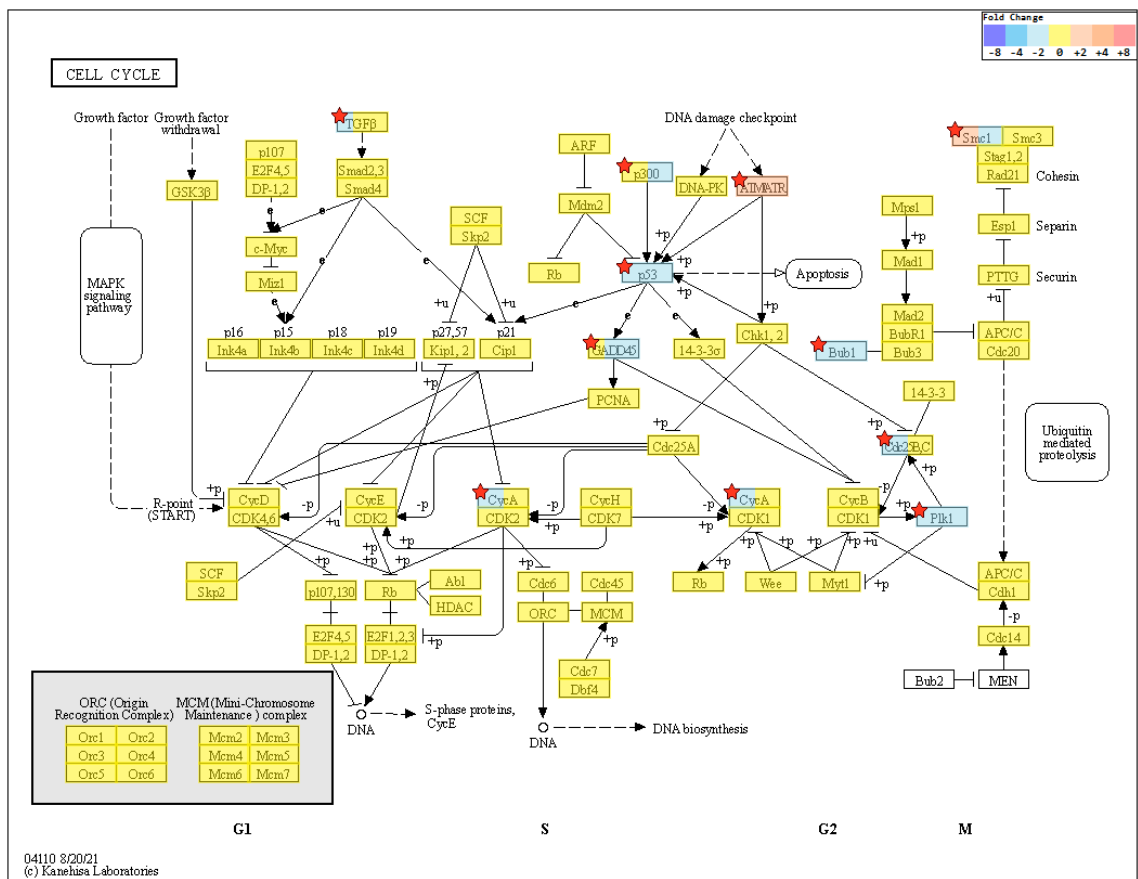

# B

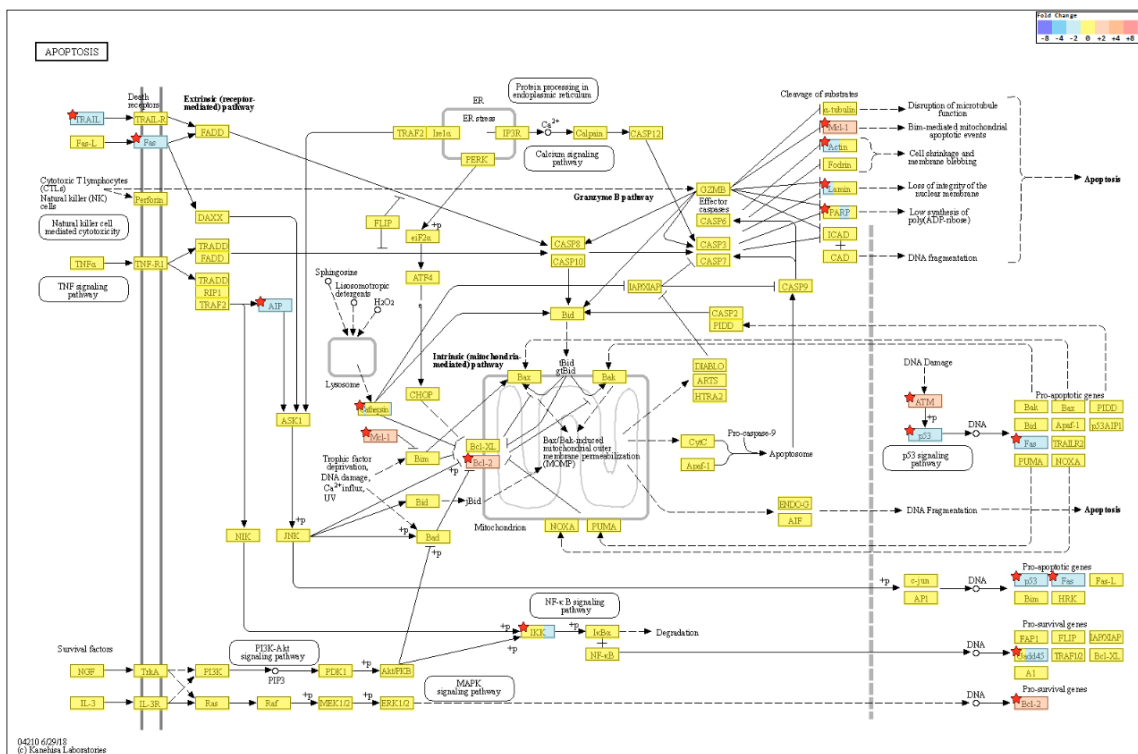

**C**

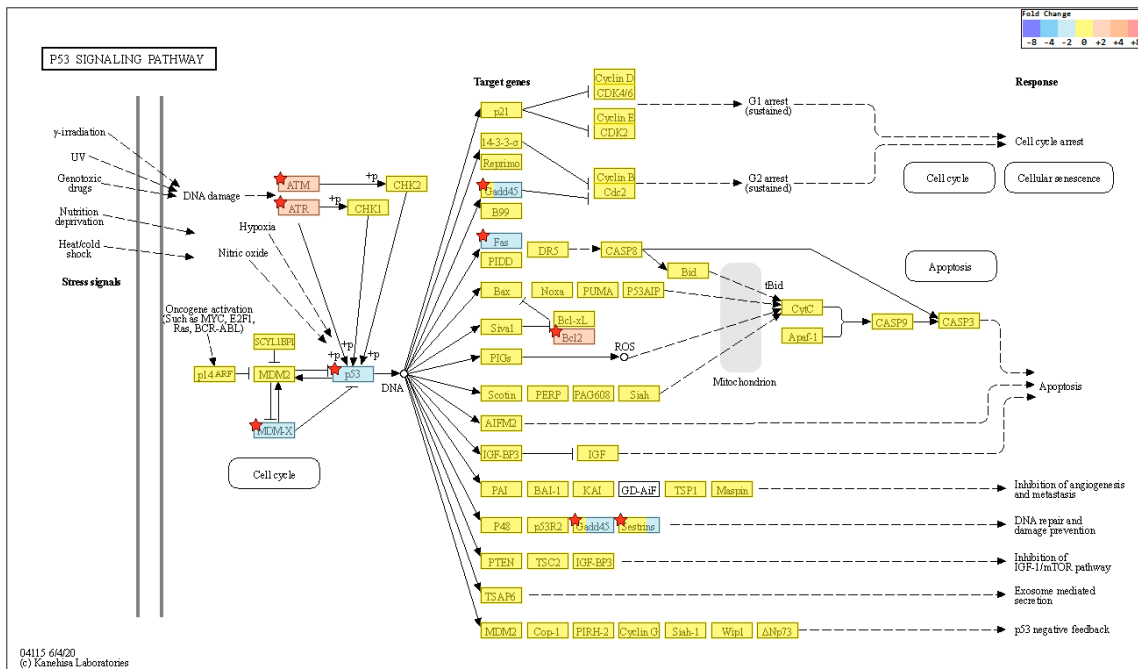

Supplement: Supplementary 1 — Figs. S1 to S3 Table S1 [file bmr.0371.f1.zip › Figure S3.pdf]
